# Enhancing Chitosan Films for Egg Packaging Using Cellulose Nanocrystals and Sodium Montmorillonite Nanoparticles

**DOI:** 10.3390/foods15060987

**Published:** 2026-03-11

**Authors:** Dariush Khademi Shurmasti, Clara Mariana Gonçalves Lima, Charles Odilichukwu R. Okpala

**Affiliations:** 1 Department of Agriculture, Sava. C., Islamic Azad University, Savadkooh GA 30602, Iran; 2 Department of Food Science, Federal University of Lavras, Lavras 37203-202, Brazil; claramarianalima@gmail.com; 3 UGA Cooperative Extension, College of Agricultural and Environmental Sciences, University of Georgia, Athens, GA 30602, USA

**Keywords:** cellulose nanocrystals (CNC), chitosan, eggshell morphology, montmorillonite (MMT), nanocomposite coating

## Abstract

Bio-based polymers are believed to often demonstrate insufficient barrier capacity and mechanical strength, especially in egg packaging processes. This current work attempted to improve the characteristics of chitosan (CS) films for egg packaging by incorporating cellulose nanocrystals (CNC) and sodium montmorillonite (MMT) nanoparticles. Such nanofillers added to the polymer matrix should reduce water vapor permeability and improve the mechanical properties of bio-nanocomposite films. Herein, coatings containing 5 wt% CNC or MMT incorporated into chitosan were applied to enhance the storability of fresh eggs over 5 weeks at ambient conditions. SEM images revealed that coatings were able to seal the eggshell pores, thereby minimizing mass transfer. After 5 weeks of storage, the Haugh unit (HU) of eggs treated with CS–CNC (67.1) and CS–MMT (64.8) appeared reasonably higher than that of control (35.2) and pure chitosan (52.1). The yolk index of eggs coated with CS–CNC (0.355) and CS–MMT (0.343) surpassed both control (0.263) and CS-coated eggs (0.308). However, pH levels in the albumen of eggs coated with CNC or MMT nanocomposite were significantly lower than others during storage. Potentially, chitosan-based nanocomposite coatings could be effective in preserving the internal quality of eggs, providing a somewhat efficient barrier against CO_2_ loss with relative pH maintenance.

## 1. Introduction

Increasingly, the demand for eggs continues to build, not only in Iran in recent years but also across several developing/developed countries, largely because eggs are an affordable source of protein. Hence, the maintenance of internal egg quality is critical, especially during the shelf storage time/supply chain from farm to fork [1]. Meanwhile, understanding the physical characteristics of the eggshell surface is equally crucial, because it helps provide clues about how microorganisms can infiltrate, as well as how gas exchanges between the inner and surrounding environment take place. With this understanding, it becomes possible to have better clarity on what leads to egg deterioration, not only from the microbiological perspective resulting in increased susceptibility to internal component degeneration, but even more so the changes in chemical properties during storage. That is why ensuring minimal chemical and biological activity specific to eggs’ outer surface is very crucial for (egg) producers [1]. Economically speaking, the ceramic structure of eggshells must be of high porous quality, and that is among the key struggles for the poultry industry.

A lot of efforts go into sealing the pathways of penetration by oils and polymers, so as to prevent mass transfer, preserve internal quality, deter/limit microbial contamination, and ultimately extend the egg shelf quality and time [2]. Meanwhile, the expected function of food bio-packaging, as it relates to the context of egg production in the poultry industry, has always been to elevate food quality and protect the environment. Achieving this has largely relied on identifying the most suitable packaging materials that would deliver the desired physical, mechanical, and barrier properties [3,4,5,6], and at the same time provide limited to zero impact on the egg’s chemical structure during the shelf time. In addition, the operational efficiency of such packaging materials has to provide room for enhancements, for instance, the ability to incorporate either organic or inorganic/synthetic particles, especially in its formulation [7]. To overcome the environmental/health risks widely understood to be associated with the use of synthetic additives/particles, however, developing biodegradable packaging materials such as chitosan has continued to gain momentum.

Specifically, chitosan [β-(1,4)-2-amino-2-deoxy-D-glucopyranose], a cationic natural carbohydrate polymer, is derived from deacetylated chitin, believed to be the second most abundant polysaccharide in nature. In recent years, the attention towards chitosan-based films has appeared considerably given their promising antimicrobial activity, biodegradability, chemical stability, eco-friendliness, excellent (film-forming) properties, and nontoxicity. However, some drawbacks of pure chitosan films appear to persist, for example their brittleness, hydrophilicity, and limited mechanical performance, which some works believe would restrict their application in food packaging [8]. That is why the incorporation of nano-level fillers as additives into the chitosan matrix could serve as an effective strategy to enhance film properties and fabricate composite materials [9,10]. Blending chitosan with other biopolymers has also been widely explored as an approach to enhance its antimicrobial and physicochemical properties. In particular, binary systems based on chitosan and starch have attracted increasing attention due to their complementary characteristics [11]. Some examples of nanofillers include montmorillonite [12] and cellulose nanocrystals [13], which appear to produce some successes in enhancing the mechanical properties of chitosan films with promising results.

Montmorillonite (MMT) is an aluminosilicate clay in which the substitution of Al ions for Si and Mg ions in its layers results in a negative surface charge on the clay. Montmorillonite has a large specific surface area and exhibits good adsorption and cation exchange properties [12]. Besides montmorillonite’s ability to improve emergent products’ tensile strength when incorporated into chitosan films [14], the addition of clay and glycerol to chitosan/clay nanocomposite films could enhance the thermal stability, tensile strength, and tensile modulus of chitosan [15]. Specifically, cellulose nanocrystals (CNCs), when charged by nanoparticles with a high aspect ratio, despite being the most common biological polymer found in plant cell walls, would express unique properties, such as a high elastic modulus and transparency [16]. Moreover, the production of chitosan-based films with varying concentrations of CNC has been shown to elevate tensile strength, water resistance and thermal stability, yet reduce water vapor permeability, of chitosan films [17]. Therefore, by immersing eggs in chitosan–polyvinyl alcohol (CS–PVA) [18] and chitosan–polyvinyl alcohol–montmorillonite (CS–PVA–MMT) [19] coating solutions, it is possible to enhance their internal quality properties and extend their shelf life during storage. It is not yet fully understood how comparing the effectiveness of nano-MMT and CNC reinforcement in chitosan films and their applications in egg preservation/storage would turn out. To supplement existing information, therefore, this current research aimed to apply organic (CNC) and inorganic (MMT) materials to ensure better integrity for incorporated nanoparticles in the CS matrix, thereby improving diverse final properties of the nanocomposites. Additionally, the efficiency of coating nanocomposite solutions was investigated to replace synthetic polymers frequently used in the packaging industry, considering factors that affect the internal quality of eggs.

## 2. Materials and Methods

### 2.1. Materials

Chitosan (Mw: 440 kDa; DD: approximately 90%) and nano-montmorillonite (MMT, K10), without organic modification, were purchased from Sigma-Aldrich Chemicals (Taufkirchen, Germany). Nano Novin Polymer Co. (Sari, Iran) provided cellulose nanocrystals (CNC). Glycerol, acetic acid, and all other reagents and chemicals were laboratory-grade (Merck, Darmstadt, Germany). Unwashed, clean, white-shelled eggs (55.8 to 60.3 g, with an average weight of 58.7 g) were purchased from a local poultry farm and were immediately used for this experiment. Importantly, all chemicals and reagents were of analytical grade standard, and procured from commercially certified retailers.

### 2.2. Film Fabrication and Characterization

The casting method described in previous studies [15,20] was applied to fabricate chitosan-based films, with some modifications. The composition of the chitosan-based coating formulations (CS, CS–CNC, and CS–MMT) is summarized in Table 1. In 100 mL of 1% (*v*/*v*) acetic acid solution, 2 g of chitosan (CS) and 0.6 g (30 wt% of CS) glycerol were added. The solution was stirred for about 2 h using a heater–stirrer (SB302, Stuart(R), Bibby Scientific UK, Staffordshire, UK) at 50 °C and 250 rpm, and then allowed to cool to room temperature. Separately, few drops of CNC or MMT (5% *w*/*w* based on CS weight) were added to 100 mL of distilled water, and dispersed under the same conditions as CS for 2 h. Then, the solutions were homogenized (IKA T25 Digital ULTRA-TURRAX Disperser, KA-Werke GmbH & Co. KG, Staufen, Germany) at 8000 rpm for 15 min. After these dispersions into the CS solution and stirring for 1 h at room temperature, the resulting solutions were cast onto the center of glass discs (8.5 cm diameter), then first dried in an oven at 37 °C for 48 h, followed by drying in ambient conditions (at 47% relative humidity) for 24 h to allow for maximum evaporation of water and residual solvents. Finally, all the dried films were peeled off and placed in hermetic plastic packaging (18 cm × 14 cm; Nano Novin Polymer Co., Sari, Iran).

### 2.3. Coating the Egg with Prepared Chitosan-Based Solutions

The selected chitosan-based coating solution was prepared similar as with the previous section. Batches of eggs were immersed separately in the recently prepared chitosan-based coating solutions for approximately 60 s using a wire dipping loop, subsequently followed by primary immersion in distilled water. The eggs were then lifted from the solution, allowed to drain, and dried under a fan for 5 min before being placed on an egg shelf and stored at ambient conditions (26 ± 2 °C, 50 ± 2% RH) for 4 weeks. Additionally, the uncoated eggs (control) were stored under the same conditions, followed by primary immersion in distilled water. At 1-week intervals, 20 specimens of coated and uncoated eggs were randomly selected to determine the internal quality parameters. At the same time, eggshells were also collected for morphological evaluations.

### 2.4. Thickness and Mechanical Properties of the Films

The film thickness was measured using a digital micrometer (Mitutoyo Company, Kanagawa, Japan) to the nearest 0.001 mm at eight random positions around the film. The mean value helped to determine the physical and mechanical properties. Tensile strength (TS), elongation at break (EB), and tensile modulus (TM) were determined using a texture analyzer (Stable Micro System, model TA-XT2, Godalming, UK) consistent with guidelines stated in the ASTM D882-18 method. Initially, the films were sliced into strips (10 × 100 mm) and placed in a desiccator at 50% RH and 25 °C for 48 h. They were then fixed with an initial grip separation of 50 mm and stretched at a specific speed (50 mm/min). TS (MPa) and EB (%) values were calculated using the following equations. The TM (MPa) value was determined from the slope of the elastic region within the stress–strain curves. TS, EB, and YM tests were replicated at least five times for each type of film.

$$\mathrm{TS}\;(\mathrm{MPa})=\frac{F_{max}}{A}$$

$$\mathrm{EB}\;(\%)=\frac{L_{2}-L_{1}}{L_{1}}$$

where F_max_, A, L_2,_ and L_1_ are the maximum load (N), initial cross-sectional area (m^2^), final length at the point of sample rupture, and initial length of the specimen (50 mm), respectively.

### 2.5. Water Vapor Permeability Measurement

The water vapor permeability (WVP) coefficient was determined gravimetrically following the ASTM E96 procedure, with a 75% relative humidity (RH) gradient at 25 °C [21]. Briefly, films (0.00287 m^2^ area) were sealed onto test vessels containing anhydrous calcium chloride desiccant, which helped to create a 0% RH storage condition. The inside RH was always lower than the outside. This RH difference corresponds to a driving force of 1753.55 Pa, which is expressed as water vapor partial pressure. Thus, the quantity of water vapor that would be transferred through the film and subsequently absorbed by the desiccant was determined by the weight gain as a function of time. The slope of weight as a function of time against the effective film area resulted in the water vapor transmission rate (WVTR). The WVP for each type of nanofilm was calculated as follows:

$$\mathrm{WVP}\;(g/m.s.\mathrm{Pa})\;=\;\frac{WVTR\;\times \;X}{∆P}$$

where WVTR is water vapor transmission rate (g/m^2^·s), X is film thickness (m), and ΔP is the vapor partial pressure difference across the film (Pa).

### 2.6. Egg Internal Quality Parameters

All egg samples were weighed to obtain an initial weight immediately before storage. Periodically, the final weights were measured during storage. From the resultant data, the average weight loss (WL) of coated and uncoated eggs was calculated by the difference between the initial and final weight, against the initial egg weight, then multiplied by 100.

The eggs were subsequently broken on a glass plate, and parameters of the Haugh unit (HU) were determined using a micrometer (AMES S-6428, Waltham, MA, USA). The calculation of HU followed the formula 100 log (H − 1.7 W^0.37^ + 7.6), where H refers to the albumen height (mm) and W refers to the egg weight (g) [22]. The expression of HU adheres to a grading/score between 0 and 100. According to the USDA egg classification manual, eggs can be classified as AA (excellent; higher than 72), A (high quality; 60–71), B (average quality; 30–59), and C (low quality; less than 29). After separating the yolk from the albumen, the yolk height was determined using a digital micrometer, whereas the yolk diameter was determined using a digital caliper (Digital INSIZE, İstanbul, Türkiye). The yolk index (YI) was obtained as YI = h/d, where h refers to the yolk height (mm), and d refers to the yolk diameter (mm) [22].

The pH of the egg albumen was measured at 25 °C using a pH meter (Eutech Instruments pH-510, Singapore).

### 2.7. Microstructural Evaluation of Eggshell Outer Surface

Eggshell samples were prepared for scanning electron microscopy (SEM) testing (SEM; AIS-2100, Seron Technology Inc., Uiwang, Republic of Korea), which enabled the microstructural evaluation of features such as uniformity, compactness, pores, roughness, ridges, bulges, and particles/granules, especially at the surface level. The SEM analysis was operated at an acceleration voltage of 27 kV and 10,000-fold magnification. The eggshell samples were placed on an aluminum stub, which was covered with adhesive tape and gold-sputtered coating. Fourier transform infrared (FTIR) spectra of the outer surface of the eggshells were recorded using an FTIR spectrophotometer (Tensor 27, Bruker Optik GmbH, Ettlingen, Germany) with attenuated total reflectance (ATR) accessories enabling spectral examination of the eggshell’s outer surfaces. FTIR analysis operated within the 400–4000 cm^−1^ spectral region with 32 scans at a resolution of 4 cm^−1^.

### 2.8. Statistical Analysis

The resulting experimental data were analyzed based on a completely randomized design. SPSS software version 20 was used to run the analyses. For all results, the mean and standard deviation (SD) values were generated from at least three independently performed experimental replicates. For statistical comparisons, both analysis of variance (ANOVA) and Tukey’s test were employed at a 95% confidence level.

## 3. Results and Discussion

### 3.1. Mechanical and Physical Film Characteristics

The thickness and mechanical properties of chitosan-based films are presented in Table 2. Incorporating the nanofiller considerably enhanced the thickness of the chitosan-based films. Similar to Ojagh et al. [21], the thickness values ranged between 95 and 115 µm, although those of CS appeared slightly lower than in data from Dehnad et al. [20], which ranged between 118 and 225 µm. Such differences in CS might be due to molecular weight discrepancies arising in processing stages.

The tensile strength (TS) of chitosan and chitosan-based composite films is also presented in Table 2. The TS of chitosan-based films revealed a range between 15.56 and 29.75 MPa, suggesting a substantial (chitosan) increase of up to 91% (*p* < 0.05), which might appear less than in previously reported data [15,23]. Differences in molecular weights within the chitosan matrix might probably be responsible, which could further attest why the thicknesses of chitosan-based film behaved as in this current study. When TS in CS–CNC increases, this might indicate stronger interfacial adhesion, specifically between CNC and the chitosan matrix, through hydrogen bonding. This should suggest an electrostatic interaction of polyelectrolyte complexes, likely between the cationic groups of chitosan and anionic sulfate groups of CNC [23]. If this were to be considered within a CS–MMT film, there could be a better stress transfer between adjacent chitosan chains, which might strengthen the electrostatic bonding between –NH_2_ and –NH_3_ groups [15]. Potentially, such nanofillers in chitosan would occupy the free space between polymer chains, thereby increasing the intermolecular attraction force.

Adding CNC to produce higher TS, as in the report of Xu et al. [24], suggests it is a fiber source that enables variations in the molecular weight [23]. Differences in TS even when adding 5 wt% MMT might connect with data shown by Kusmono and Abdurrahim [15]. The tensile modulus (TM) of the chitosan film of this current work was 1630 MPa. Adding 5 wt% CNC or MMT into the chitosan matrix significantly increased the TM by 107% and 93.2%, respectively. Such an increase in the TM might suggest stiffer CNC particles [17]. Enhancing TM for MMT loading may help reinforce the silicate layers of clay, which might better integrate within the chitosan matrix and be able to elevate the aspect ratio and surface area, with better interactions between the polymer matrix and hydrogen bonds [15]. The elongation at break (EB) of chitosan film showed a value of 14.30%, but significantly decreased nanofillers were added (*p* < 0.05). Nevertheless, tensile modulus (TM) showed an increase of 107% when CNC was incorporated, but a decrease of 253% in elongation at break (EB) compared to pure chitosan (Table 2). Possibly, a continuous network of cellulosic nanoparticles might have formed and linked through hydrogen bonding [25]. Other authors, like Souza et al. [26], found similar results when CNC and MMT were added to a pectin matrix. Montmorillonite might not necessarily improve the mechanical properties of films as much as CNC. The nanofiller type is very crucial and can influence the properties of films and exert small differences in the mechanical behavior of bionanocomposites. Moreover, the nature of NPs such as MMT, as inorganic with platelet-like morphology, and CNC, as organic with fiber morphology, would provide different interactions and produce polymers of diverse structural and arrangement features [27].

Water vapor permeability (WVP) is critical specifically in edible films, especially in the context of food product shelf time. Table 2 shows WVP (×10^−10^ g·m/(m^2^·s·Pa)) significantly decreased with added nanofillers within the polymeric matrix, from 3.85 for pure chitosan film down to 2.30 and 2.65 for 5 wt% loading of CNC and MMT, respectively (*p* < 0.05). Possibly, CNC might have had a slightly better impact compared to MMT in reducing the WVP values of chitosan-based films. A lower WVP value suggests reduced moisture exchange between the food and the surrounding atmosphere, which can help prevent or slow down the progress of food deterioration [28]. This may be attributed to the better dispersion of nanoparticles in the chitosan matrix. The hydrophilic or hydrophobic nature of materials, along with factors such as the type, level, and dispersion quality of additives, including pores, cracks, and chain order of the polymer, have their role in water vapor permeability/water vapor transmission rate of films [20]. The presence of nanofillers should make water molecules unable to diffuse into the composites, thereby decreasing the water permeability [29]. Previous authors also believed a reduction in the WVP of nanocomposite films would arise by adding CNC [17,20] and MMT [12].

### 3.2. Morphological Properties of Coated Eggshells

**Figure 1:**
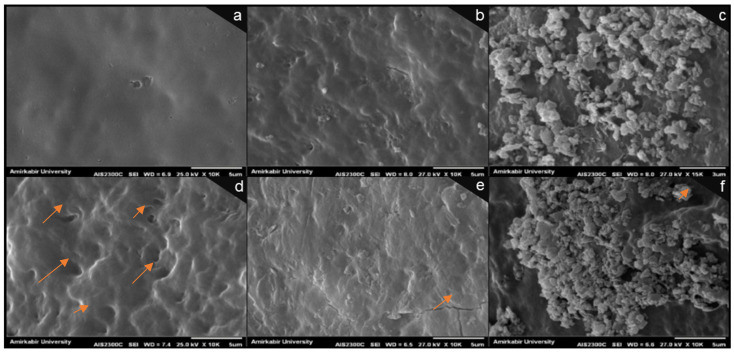
SEM images of the eggshell surface with different coatings: the images in the first row are from day 0, and the second row corresponds to day 35. CS coating (**a**,**d**), CS–CNC coating (**b**,**e**), and CS–MMT coating (**c**,**f**) were imaged using magnification of 10,000 and a scale bar of 5 μm. CS: chitosan; CNC: cellulose nanocrystal; MMT: montmorillonite. Arrows show some micro-pits, wrinkles, and discontinuities in images.

The coating of eggshells should seal the (shell) pores by forming barrier layers. This should provide relatively effective protection for the eggs, which helps extend the shelf life. Hence, to evaluate the surface morphology of the coated eggshells, SEM analysis was performed at 0 and 35 days. SEM images of the samples’ surface morphology are shown in Figure 1. As demonstrated in Figure 1a, the morphology of the surface of chitosan films appears rather homogeneous and smooth. However, the incorporation of CNC into chitosan films seemed to produce a rougher surface, which tended to be more pronounced when MMT was added. Moreover, a uniform distribution of the nanofillers seems somewhat apparent, specifically within the chitosan matrix (Figure 1b,c). Potentially, the acetic acid that was used in the preparation of coatings of this current study might have sealed the primary micro-cracks by reacting with eggshell calcium carbonate [30].

If, for any reason, the outer surface structure of the eggshell were to be destroyed, even if including environmental/external factors, the barrier properties are more likely to weaken equally [30]. In this current work, the SEM images across all of Figure 1a–f exhibit a typical trend that suggests differences in the structure of nanocomposite-coated eggshells could emerge compared to that of pure chitosan ones. For instance, on day 35, some kinds of micro-pits, wrinkles, and discontinuities appeared, which tended to be more pronounced on the chitosan-coated eggs compared to the CNC and MMT nanocomposite coatings. It is more likely that the incorporation of nanofiller into chitosan-based coatings brought about a rather new, probably different morphology, specifically on the surface of the eggshells. Demonstrably, Figure 1e,f suggests lower surface porosity in the SEM images of nanocomposites compared with those of pure chitosan coatings, most likely attributable to uniform nanofiller distribution within the chitosan matrix. In this context, CNC should be better distributed within the chitosan polymer and if so, can more effectively maintain its primary structure during storage, compared to MMT.

**Figure 2:**
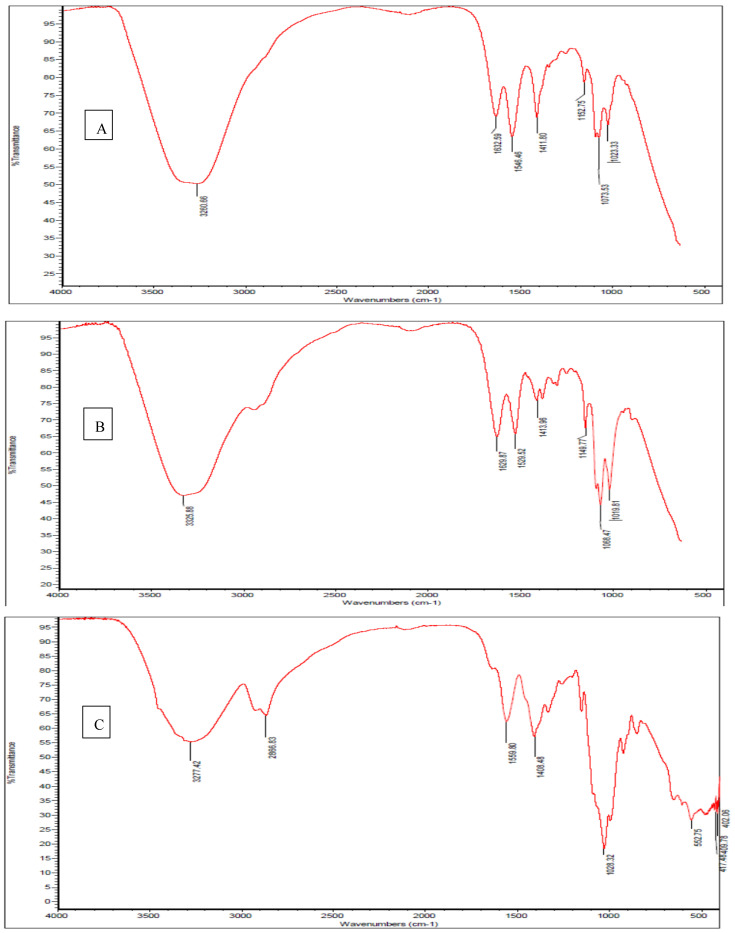
FT-IR spectra of eggshell coating based on CS (**A**), CS–CNC (**B**), and CS–MMT (**C**); CS: chitosan, CNC: cellulose nano crystal; MMT: montmorillonite.

To deepen our understanding of nanofiller effects on chitosan-based coating/film on eggshells in this current study, FTIR spectra helped reveal some structural features, including potential interactions among the outer surface components. Figure 2 shows the FT-IR spectra of eggshell coating based on CS (Figure 2A), CS–CNC (Figure 2B), and CS–MMT (Figure 2C; where CS: chitosan; CNC: cellulose nano crystal; MMT: montmorillonite). The chitosan coating spectrum (Figure 2A) exhibited a broad band typical of this biopolymer, specifically located at 3260 cm^−1^, which can be assigned to O–H and N–H stretching vibrations. Moreover, the bands specifically located at 1632, 1546, and 1411 cm^−1^ can be respectively assigned to carbonyl group (C=O) stretching (amide I), amino group (NH_2_) bending (amide II), and the carboxyl group (–COO–). Furthermore, the bands specifically located at 1152 and 1023 cm^−1^ can be respectively assigned to saccharide structure stretching of the C–O–C bond, and C–O stretching vibrations [12].

FTIR results further revealed somewhat of an influence of CNC integration on the chitosan coating, which seems to reflect how the infrared bands are characterized as well as shifted by CNC nanocomposite interactions. For instance, the absorption peaks of the CNC nanocomposite coating at 3500–3250 cm^−1^ would likely account for the stretching of intramolecular O-H and CH_2_OH vibrations, despite the observations of the Amide I vibrational mode at 1629 cm^−1^ and Amide II at 1529 cm^−1^. Authors like Li and Renneckar [31] understood sharp peaks that happen at 3325 cm^−1^ can likely be attributed to O–H vibrations arising from intramolecular hydrogen bonding. Possibly, such C–O–C stretching at 1149 cm^−1^ should therefore be associated with the absorption band. Additionally, the CNC nanocomposite spectrum at 1068 and 1019 cm^−1^ might appear as stronger bands given by C–O stretching at the C-3 position [13], which in this current work seem to be demonstrated in the FTIR spectra of the chitosan coating in Figure 2B, caused by the incorporation of CNC into the chitosan matrix. Moreover, distinct peaks seemed absent in the pure chitosan coating spectrum at 3325 cm^−1^. Furthermore, the intensity of the band at 3342 cm^−1^ is higher, suggesting the existence of hydrogen bonds between chitosan and CNC, in agreement with Khan et al. [17].

The FT-IR spectra of MMT nanocomposite coating revealed absorption peaks at 3277 cm^−1^ for N–H stretching and at 2866 cm^−1^ for C–H stretching in –CH_3_. In addition, the pure chitosan coating with peak at 1559 cm^−1^ can be assigned to N–H bending (amide II), while the peak at 1408 cm^−1^ can be assigned to C–N stretching (amide III) [32]. The FT-IR spectra of MMT nanocomposite coating (Figure 2C) peaked at 1028 cm^−1^, which can be attributed to the Si–O–Si vibration, possibly indicative of clay present, as reported by Kusmono and Abdurrahim [15].

### 3.3. Internal Changes in Egg Quality

**Figure 3:**
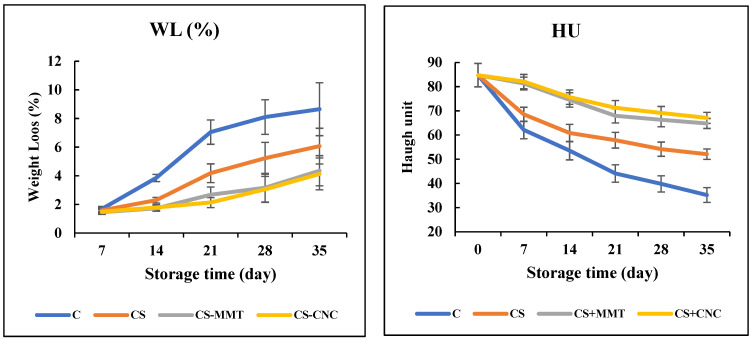
Weight loss (WL; %) and Haugh unit (HU) changes in the eggs during ambient storage (35 days); C: control (uncoated eggs); CS: chitosan, CNC: cellulose nanocrystal; MMT: montmorillonite. Values are expressed as mean ± standard deviation (*p* < 0.05).

According to Xu et al. [30], the main contributors to egg weight loss (WL), which significantly affects (egg) quality evaluation, include water evaporation and carbon dioxide released from the albumen, passing through the porous shell into the external environment. In the current work, Figure 3 shows all egg samples had WL gradually increase with storage time, more so in uncoated than in CS-coated eggs (*p* < 0.05). After 2 weeks of storage, WL appeared to resemble that of coated eggs. However, a positive effect was detected in the nanocomposite coating beyond weeks 3–5 compared to other groups (*p* < 0.05).

The Food and Agriculture Organization of the United Nations (FAO) states that a reduction in egg weight by 2% to 3% while storing is acceptable. All nanocomposite-coated eggs maintained WL that appeared acceptable in week 3, whereas the uncoated control would only be acceptable at week 1. Despite a decrease in average weight by a little over 3%, 3.05% in CNC and 3.16% in MMT, the nanocomposite-coated eggs remained acceptable after week 4. A similar week 4 outcome was achieved by Pham et al. [33], who specifically employed a starch coating that limited the transmission of gas exchange and moisture to the environment (*p* < 0.05). It is possible that the effectiveness of the coating to strengthen shelf time could be increased by adding MMT or CNC to a CS matrix.

In the poultry industry, Haugh unit (HU) is one of the most widely used egg quality parameters. The higher the HU value of an egg, the better the albumen quality. Figure 3 shows that the HU of egg samples decreased over the storage period. However, coated eggs showed a significantly lower reduction in HU compared to the control samples.

Table 3 shows the grade of the eggs over 5 weeks of storage based on HU. All egg samples maintained their A grade in the first week. The CS-coated samples remained A grade until the second week, while the nanocomposite-coated eggs maintained A grade up to the fifth week. The results of HU agreed with the results of WL (Figure 3), demonstrating that the coating could improve the shelf life of eggs by up to 3 weeks at room temperature. The reduction in HU is associated with moisture migration from albumen to yolk during the storage period, which is the main reason for albumen viscosity changes [34]. Additionally, pores on the eggshell surface lead to the diffusion of CO_2_ and moisture, resulting in a reduction in HU. The reason for this behavior may be due to the association of ovomucin with lysozyme, disulfide bonds, carbohydrate moieties of ovomucin, or the relationships between α and β ovomucins [33].

**Figure 4:**
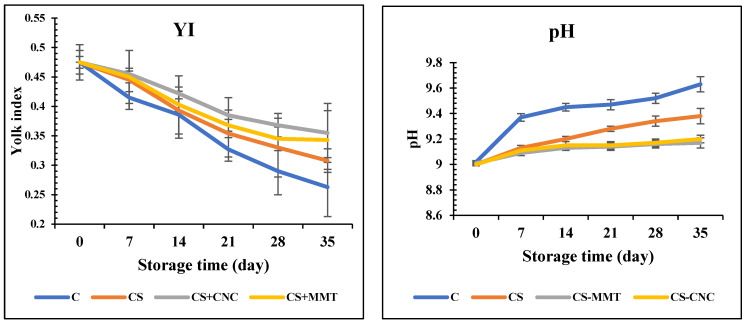
Yolk index (YI) and pH changes in the eggs during ambient storage (35 days); C: control; CS: chitosan, CNC: cellulose nanocrystal; MMT: montmorillonite. Values are expressed as mean ± standard deviation (*p* < 0.05).

The yolk index (YI) is an indicator of the internal quality of table eggs. Similar to the result of HU, both coated and control samples showed a decline in YI, but the reduction in uncoated eggs was more dramatic (Figure 4). The YI values for the control and eggs coated with CS, CS–MMT, and CS–CNC dropped from 0.475 to 0.273, 0.308, 0.355, and 0.343 after 5 weeks of storage, respectively. This could be attributed to the coating acting as a barrier, preventing the release of CO_2_ and moisture into the environment. The conclusions reached are in line with Pham et al. [33].

YI can also be used to indirectly measure the strength of the yolk vitelline membrane, which helps evaluate egg freshness. Therefore, the higher the YI, the better the yolk quality. In the current work, the YI of eggs coated with chitosan-based nanomaterials was noticeably higher than that of uncoated eggs, especially after week 5 (*p* < 0.05). A decrease in YI indicates a weakening of the yolk vitelline membrane, which separates the yolk from the albumen [35].

To assess egg freshness and quality, the pH value is very crucial. The breakdown of the thick albumen and the hydrolysis of carbonic acid inside the eggs release CO_2_ through the shell pores [33]. The denaturation of proteins might elevate the pH and decrease the HU [36]. As exhibited in Figure 4, after 5 weeks of storage, the pH of albumen significantly differed between control samples (pH from 9.02 to 9.63) and coated samples (pH from 9.00 to 9.17–9.38). The albumen pH range for freshly laid eggs is usually 6.0–7.7, but can quickly increase to 9.0–9.7 due to CO_2_ emission [36]. The coating effectively seals pores and acts as a barrier against CO_2_, thereby regulating albumen pH and strengthening egg quality [33].

Table 4 shows the correlation between the mechanical and physical characteristics of the film/coating with egg internal quality properties. A clear correlation exists between the film’s mechanical and physical characteristics, as well as with egg internal quality properties. Thickness positively correlates with TS (r = 0.87) but negatively correlates with EB and WVP. Meanwhile, TS aligns positively with egg HU, YI, while negatively with pH and WVP. Finally, WVP relates severely negatively to HU and YI, yet positively to WL. Accordingly, the higher HU and YI, and lower WL, values in eggs with CS-based coatings can be attributed to decreased WVP.

## 4. Conclusions

The investigation focused on using chitosan coatings and adding CNC and MMT as nanomaterials to maintain fresh egg qualities, particularly functional characteristics. The effectiveness of these coatings lies in their ability to maintain internal qualities and reduce food losses by sealing pores on the shell to reduce mass transfer. Sealing pores on the eggs’ surface directly affects the mass transfer properties of coated eggshells. The results indicate the films manufactured based on chitosan have favorable mechanical and physical properties (based on TS, TM, EB and WVP), and the prepared coating solutions could be a favorable alternative for preserving the functional characteristics of the eggs (based on pH, HU and YI during storage) in industrial use, especially once supplemented with nano- organic and inorganic filler. Chitosan-based nanocomposite coatings are emphasized in this study because they can maintain their quality for at least 2–3 weeks longer than control ones. Hence, chitosan-based coatings have the potential to lessen the dependency on supply chain facilities without compromising the quality of fresh eggs. The use of chitosan coatings could assist retail sectors in minimizing economic losses caused by breakage or cracks, and also alleviate many of the problems faced within various foods. The effectiveness of chitosan–CNC was superior to that of the control or pure chitosan. The shell’s strength was preserved while bio-nanocomposite coatings covered the micro-cracks and holes. Future research is needed on different coating materials containing nanoparticles and natural antimicrobials in various perishable foods. Future studies should focus on consumer acceptance and the antibacterial effect of chitosan-based nanocomposite coatings.

## Figures and Tables

**Table 1:** Composition of chitosan-based coating formulations.

Film Formulation	Composition
CS	chitosan (2 g) and glycerol (0.6 g; 30 wt% of CS) dissolved in 100 mL acetic acid 1% *v*/*v* (CS solution)
CS–CNC	CNC (5% *w*/*w* CS) dissolved in 100 mL of distilled water (CNC solution) was added to the CS solution
CS–MMT	MMT (5% *w*/*w* CS) dissolved in 100 mL of distilled water (MMT solution) was added to the CS solution

**Table 2:** Mean thicknesses and mechanical properties of chitosan-based films.

Films	Thickness (μm)	Mechanical Properties			WVP × 10^−10^ (g·m/(m^2^·s·Pa))
		Tensile Strength (MPa)	Tensile Modulus (MPa)	Elongation at Break (%)	
CS	97 ± 0.003 ^b^	15.56 ± 1.97 ^c^	1630 ± 117 ^b^	14.30 ± 0.85 ^a^	3.85 ± 0.157 ^a^
CS + CNC	113 ± 0.002 ^a^	29.75 ± 2.15 ^a^	3375 ± 263 ^a^	4.05 ± 0.45 ^b^	2.30 ± 0.075 ^b^
CS + MMT	115 ± 0.003 ^a^	24.13 ± 2.86 ^b^	3150 ± 220 ^a^	4.95 ± 0.81 ^b^	2.65 ± 0.105 ^b^

Different letters in the same column correspond to statistically different samples (*p* < 0.05). CS: chitosan; CNC: cellulose nanocrystals; MMT: nano-montmorillonite; WVP: Water Vapor Permeability.

**Table 3:** Grade of the eggs during five weeks of storage based on HU.

Treatments	Storage Time (Day)					
	0	7	14	21	28	35
C	AA	A	B	B	B	B
CS	AA	A	A	B	B	B
CS + CNC	AA	AA	AA	A	A	A
CS + MMT	AA	AA	AA	AA	A	A

C: control, CS: chitosan, CNC: cellulose nanocrystal, MMT: montmorillonite.

**Table 4:** Correlation between mechanical and physical characteristics of film/coating with egg internal quality properties.

	Thickness	TS	EB	WVP	WL	HU	YI	pH
Thickness	1							
TS	0.874846	1						
EB	−0.98368	−0.94772	1					
WVP	−0.9497	−0.98253	0.990547	1				
WL	−0.97889	−0.95538	0.999689	0.99366	1			
HU	0.970314	0.966027	−0.99799	−0.99725	−0.99926	1		
YI	0.939449	0.987872	−0.98577	−0.99951	−0.98966	0.994439	1	
pH	−0.99952	−0.85945	0.97765	0.939569	0.972103	−0.96237	−0.92841	1

TS: Tensile Strength; EB: Elongation at Break; WVP: Water Vapor Permeability; WL: Weight Loss; HU: Haugh Unit; YI: Yolk Index.

## Data Availability

The original contributions presented in this study are included in the article. Further inquiries can be directed to the corresponding authors.
